# Inflammation: a key mechanism connecting metabolic-associated steatotic liver disease and systemic arterial hypertension

**DOI:** 10.3389/fimmu.2025.1620585

**Published:** 2025-09-05

**Authors:** Helena Solleiro-Villavicencio, Rebeca Viurcos-Sanabria, José Alfredo Aguayo-Guerrero, Pablo Fernando Pineda-Pérez, Lucía Angélica Méndez-García

**Affiliations:** ^1^ Posgrado en Ciencias Genómicas, Universidad Autónoma de la Ciudad de México, Mexico City, Mexico; ^2^ Licenciatura en Ciencias Genómicas, Universidad Autónoma de la Ciudad de México, Mexico City, Mexico; ^3^ Department of Plastic and Hand Surgery, Medical Center-University of Freiburg, Freiburg, Germany; ^4^ Laboratory of Immunometabolism, Research Direction, General Hospital of Mexico “Dr. Eduardo Liceaga”, Mexico City, Mexico

**Keywords:** MASLD, SAH, chronic inflammation, cytokines, chemokines, liver disease, metabolism

## Abstract

Metabolic dysfunction-associated steatotic liver disease (MASLD) is the most common chronic liver condition worldwide. The increase in the prevalence of MASLD is linked to the global rise in obesity. MASLD encompasses a disease spectrum beginning with simple steatosis that may progress to metabolic dysfunction-associated steatohepatitis (MASH), cirrhosis, and hepatocarcinoma. Clinical studies highlight the bidirectional relationship between MASLD and systemic arterial hypertension (SAH), showing that MASLD patients have a higher risk of developing SAH. Likewise, hypertensive patients show an increased susceptibility to MASLD, suggesting mutual pathogenic mechanisms. Inflammation is a shared pathway between these two entities; MASLD pathogenesis encompasses hepatic lipotoxicity, inducing the release of pro-inflammatory mediators, which promote systemic inflammation, contributing to vascular remodeling, increased blood pressure, and deregulating the renin-angiotensin system (RAS), potentially contributing to SAH. On the other hand, chronic hypertension promotes hepatic inflammation through immune and neuroendocrine pathways, favoring progression from MASLD to MASH. This review, emphasizing the pro-inflammatory factors, explores the inflammatory crosstalk between MASLD and SAH. Understanding this interplay provides a comprehensive perspective on chronic inflammation that could link liver and vascular pathologies, offering potential therapeutic targets for treating both conditions.

## 1 Introduction

Metabolic dysfunction-associated steatotic liver disease (MASLD), formerly known as nonalcoholic fatty liver disease (NAFLD), stands as the most prevalent liver condition on a global scale (1). The factors contributing to the escalating incidence of MASLD are urbanization, unhealthy dietary patterns, and sedentary lifestyles. This condition begins with the accumulation of fat within hepatocytes, a stage referred to as steatotic liver disease (MASL). This intracellular lipid accumulation primarily results from increased *de novo* lipogenesis and the uptake of circulating free fatty acids (2). As the disease progresses, lipotoxicity and inflammatory injury to hepatocytes may develop, leading to metabolic dysfunction–associated steatohepatitis (MASH), characterized by the presence of inflammation and/or fibrosis (3).

Over the last three decades, the global prevalence of MASLD has increased from 25.3% to 38%, and this is related to the pandemic of obesity and type 2 diabetes mellitus (T2DM) (1). Therefore, MASLD is associated with other metabolic diseases, and it is primarily prevalent within approximately 70% of individuals with T2DM. Moreover, the presence of MASLD is also associated with metabolic comorbidities, such as cardiovascular disease (CVD) and systemic arterial hypertension (SAH), defined as having a systolic blood pressure (BP) over 130 mmHg or a diastolic BP exceeding 80 mmHg (4).

The connection between MASLD and SAH has been well-documented (5). Prospective clinical studies have revealed that individuals with MASLD are more likely to develop SAH compared to those without this liver condition. Specifically, “adjusted hazard ratios for prehypertension and hypertension are higher in individuals with mild MASLD (1.18 and 1.07, respectively) and even greater among individuals with moderate-to-severe MASLD (1.62 and 1.14, respectively)” (6).

Conversely, SAH also predicts the development of MASLD. Cohort and prospective clinical studies have shown that the onset and persistence of hypertension increase the risk of MASLD, with odds ratios of 1.45 and 1.61, respectively. However, the exact causal relationship between the two conditions remains debated, as they can mutually influence each other’s development and progression (6).

The bidirectional relationship between MASLD and SAH is partially mediated by inflammation. In MASLD, hepatocyte injury and lipotoxicity trigger immune responses that increase the levels of pro-inflammatory factors (7), such as interleukin (IL)-6 and CC-chemokine ligand 2 (CCL2), contributing to chronic inflammation and stimulation of the sympathetic nervous system (SNS), which can increase BP. In parallel, inflammatory signals can also activate the RAS, a key regulator of BP. Ang-2, the main effector of RAS, promotes IR by impairing insulin receptor signaling in the liver and further contributes to hepatic injury and fibrogenesis. Notably, a genetic variant in the Ang-2 type 1 receptor (AT1R) has been associated with an increased risk of developing MASLD (5, 6).

Patients with MASLD frequently exhibit cardiac dysfunction and elevated levels of circulating inflammatory cytokines, including IL-18 and tumor necrosis factor alpha (TNF-α) (6). Moreover, recent studies suggest that T helper 2 (Th2)–derived cytokines, particularly IL-4 and IL-13, may modulate hepatic RAS activity and contribute to the development of SAH in individuals with MASLD and obesity (8).

In this article, we examine the inflammatory mechanisms that link MASLD and SAH, aiming to enhance our understanding of their interrelationship and identify potential therapeutic targets.

## 2 Pathogenesis of MASLD

MASLD is a spectrum of liver conditions that range from MASL to MASH. The pathogenesis of this disease is explained by the “multiple hit” hypothesis, which proposes that various hepatic and extrahepatic factors interact in genetically susceptible individuals to cause metabolic liver injury (9). Moreover, the development and progression of MASLD involve the dysregulation of cellular mechanisms, such as endoplasmic reticulum stress, oxidative stress, autophagy, inflammation, and apoptosis, which are triggered by risk factors that include obesity, IR, hypertension, and hypertriglyceridemia (10).

Lipid metabolism disorders cause liver fat accumulation (steatosis) (11), a hallmark of MASLD. For instance, consuming saturated fats and simple carbohydrates, the breakdown of white adipose tissue, and the liver’s fat production leads to steatosis (12). Free fatty acids (FFAs) are broken down in the mitochondria through beta-oxidation to form triglycerides (TGs). Notably, the liver and adipose tissue are closely linked because fatty acids are released from fat stores into the bloodstream and transported to the liver. Proteins such as fatty acid transport protein (FATP) 2 and FATP5 contribute to this transport process. Studies have shown that a deficiency in these FATPs can reduce hepatic fatty acid synthesis, as observed in animal models with high-fat diets (HFD). These results indicate the significance of these transport proteins in the development of liver steatosis. In patients with MASLD, fatty acid translocase CD36 levels are increased on hepatocyte membranes. Key enzymes involved in *de novo* lipogenesis (DNL), such as acetyl-CoA carboxylase (ACC) and fatty acid synthase (FAS), are elevated in MASLD patients, further demonstrating the importance of DNL in lipid accumulation. Although the exact mechanisms behind DNL activation in MASLD are still unknown, its breakdown is considered a critical factor in developing liver steatosis (11, 12).

Moreover, elevated levels of TG in the bloodstream can lead to abnormal fat accumulation and cellular dysfunction in non-adipose tissues. This is due to increased FFAs oxidation, causing mitochondrial dysfunction and the production of excessive reactive oxygen species (ROS), which contribute to an oxidative stress state that leads to liver disease. Lipotoxicity and organelle damage in MASLD play a crucial role in hepatocyte death through inflammatory pathways, endoplasmic reticulum (ER) stress, and inflammatory vesicle activation; therefore, they are critical to the development of MASH (11, 12).

IR is considered the critical pathophysiological factor in MASLD. IR increases the breakdown of fats in adipose tissue (lipolysis), which raises FFAs in the bloodstream and reduces glycogen storage in the liver (13). This promotes gluconeogenesis in MASLD patients. Hyperinsulinemia, a response to systemic IR, enhances DNL in the liver, leading to increased intrahepatic lipid accumulation. These lipids are secreted as very low-density lipoproteins (VLDL) and transported to adipose tissue, impairing fat storage. Lipotoxicity disrupts insulin signaling, causes oxidative damage, and triggers inflammation and fibrosis, contributing to the progression of MASLD (13).

In addition to lipid metabolism abnormalities, inflammation contributes to IR. Overexpression of pro-inflammatory cytokines and transcription factors in adipose tissue and the liver is observed. Obesity, characterized by chronic low-grade inflammation, is a primary cause of decreased insulin sensitivity and a significant risk factor for both IR and MASLD. Obesity-induced lipid accumulation activates signaling pathways, increasing pro-inflammatory cytokines such as tumor necrosis factor-alpha (TNF-α) and IL-6 (11, 13).

The upcoming sections will analyze the inflammatory mechanisms underlying the development and progression of MASLD and their potential role as a link between this disease and SAH.

### 2.1 Role of inflammation in MASLD

Maintaining immune homeostasis within the liver is crucial for optimal physiological function; conversely, its disruption is intricately linked to obesity and the progression of MASLD (Petrescu et al., 2022). Recent research introduces a three-stage model: 1) recognition of hepatic stress by local immune cells and subsequent cytokine release; 2) amplification of inflammatory response by innate immune cells; and 3) tissue damage driven by adaptive immune mechanisms (3).

#### 2.1.1 Early inflammatory response and innate immunity

In the initial phase, hepatic stress signals —triggered by lipid accumulation—activate tissue-resident innate-like T cells, particularly γδ T cells that produce interleukin-17A (IL-17A) (14). These cells respond to lipid-derived antigens from the gut microbiota and metabolic stress in hepatocytes. Cholesterol accumulation, for example, induces hepatocytes to express stress ligands such as MHC class I polypeptide-related sequence A/B (MICA/MICB), which interact with the natural killer group 2 member D (NKG2D) receptor. Experimental models show that a deficiency in IL-17A or cluster of differentiation 1d (CD1d) results in attenuated liver inflammation and fibrosis (3).

Activation of innate immunity leads to the release of cytokines such as IL-17A, which stimulates hepatocytes, Kupffer cells (KC), and hepatic stellate cells (HSCs) to produce chemokines that attract circulating myeloid cells (3, 14, 15). Liver sinusoidal endothelial cells (LSECs) upregulate adhesion molecules like vascular cell adhesion molecule 1 (VCAM-1) to facilitate immune cell infiltration. Neutrophils enter the hepatic tissue and release neutrophil extracellular traps (NETs), while eosinophils contribute by producing IL-13 (Fang et al., 2024). Additionally, platelets amplify inflammation via IL-1β secretion (16), and hepatocytes under lipotoxic stress release damage-associated molecular patterns (DAMPs), which further stimulate innate immune cells (3, 17, 18).

MASLD has been classified as a type 3 inflammatory disease, marked by IL-17A–mediated responses and fibrosis (15). During the early stages, this inflammation coexists with attempts at tissue repair, such as macrophage-mediated clearance and resolution (18).

#### 2.1.2 Transition to adaptive immunity and progression to MASH

As inflammation persists, adaptive immune cells infiltrate the liver, contributing to the transition from MASLD to MASH. CD4+ T helper 17 (Th17) cells, cytotoxic CD8+ T cells, and B cells promote chronic inflammation, hepatocyte apoptosis, and extracellular matrix deposition by activated HSCs—hallmarks of progressive fibrosis (3).

The activation of these immune cells is contingent upon an inflammatory environment established by myeloid cells and type-1 dendritic cells (cDC1). In animal models of MASH, elevated levels of C-X-C motif chemokine ligand (CXCL)-9 and CXCL-10 in the liver attract Th17 cells through the CXC receptor-3 (CXCR-3), thereby intensifying inflammation (19). The inhibition of these chemokines or the disruption of CXCR3 function on T cells has been shown to mitigate liver damage in experimental settings.

#### 2.1.3 Adaptive immune response and progression to severe MASLD

In advanced stages of liver inflammation, MASH arises as adaptive immune cells, including CD4^+^Th17, CD8^+^T, and B cells, which infiltrate the liver and contribute to the progression of the disease. This phase is characterized by significant hepatocyte apoptosis and the accumulation of extracellular matrix by activated HSCs, potentially leading to irreversible fibrosis (3). The activation of these immune cells is contingent upon an inflammatory environment established by myeloid cells and type-1 dendritic cells (cDC1). In animal models of MASH, elevated levels of C-X-C motif chemokine ligand (CXCL)-9 and CXCL-10 in the liver attract Th17 cells through the CXC receptor-3 (CXCR-3), thereby intensifying inflammation (19). The inhibition of these chemokines or the disruption of CXCR3 function on T cells has been shown to mitigate liver damage in experimental settings.

The role of B cells in MASLD is under investigation; however, emerging evidence indicates their involvement in the progression of the disease. Studies conducted on MASLD patients and animal models of the disease have reported increased populations of B cells within the liver. Observations indicate that some patients with MASLD exhibit elevated levels of circulating antibodies, including immunoglobulin A (IgA), immunoglobulin M (IgM), and immunoglobulin G (IgG), with higher concentrations of IgG specifically targeting oxidative stress-related antigens (anti-OSE IgG) (7). Furthermore, murine models of MASLD demonstrate that the depletion of IL-10-producing regulatory B cells has an adverse effect on immune regulation. IgA, derived from metabolically active intestinal B cells, also contributes to liver inflammation by activating myeloid cells (3).

Resident liver parenchymal and immune cells also produce immune mediators, which collectively activate both the innate and adaptive immune systems, promoting the development and progression of MASLD.

Overall, the progression of MASLD involves interactions between hepatic cells and immune populations mediated mainly by cytokines like IL-17A, IL-1β, TNF-α, and IL-13. These signals recruit immune cells, influence hepatocytes, promote tissue damage, and drive the progression of fibrosis. Understanding this molecular crosstalk is crucial for identifying therapeutic targets to halt disease progression.

## 3 Epidemiology and pathophysiological basis of SAH

Chronic high BP in the systemic arteries is the hallmark of SAH. It is recognized as the most crucial modifiable risk factor for global mortality and morbidity (20). Worldwide, approximately 1.28 billion adults aged 30 to 79 suffer from this disease. Alarmingly, nearly 46% of those affected do not know they have it. The majority of those diagnosed have essential or primary SAH, indicating that the exact cause is unknown. Conversely, about 10% of cases are classified as secondary hypertension, where a specific cause can be identified (for example, primary aldosteronism, pheochromocytoma, or renal artery stenosis) (21).

SAH is a complex disease resulting from various physiological systems, genetic factors, and environmental influences, such as high sodium intake, inadequate sleep quality, high alcohol consumption, and elevated mental stress. Furthermore, the likelihood of hypertension increases with age due to arterial stiffening, which is linked to vascular collagen changes and atherosclerosis (20, 22).

Positive family history is frequently observed among individuals with hypertension, and heritability estimates range from 35% to 50% in most studies. Genome-wide association studies (GWAS) have identified approximately 120 *loci* linked to blood pressure regulation, contributing to roughly 3.5% of the trait variance (22).

BP maintenance depends on the interactions of various neuroimmunoendocrine systems, including the RAS, natriuretic peptides, the endothelium, the SNS, and the immune system. Dysregulation of any of these factors can lead to elevated BP, either directly or indirectly. Eventually, this can lead to damage to target organs, including left ventricular hypertrophy and chronic kidney disease (CKD), which increases the risk of adverse CVD outcomes (22).

The RAS regulates BP and hydroelectrolytic balance. Classically viewed solely as a circulating endocrine system, however, recent research has identified local RAS components in organs such as the kidney, lung, and liver. Discovering local RAS components emphasizes their potential role in disease progression or protection, depending on the organ and physiological context. These systems operate through autocrine, paracrine, and intracrine mechanisms, influencing growth, differentiation, proliferation, apoptosis, oxidative stress, inflammation, fibrosis, and hormone secretion (23–25).

RAS regulation includes two pathways with opposing effects: the classical pathway, in which renin cleaves angiotensinogen to form Ang-1, which is then converted to Ang-2 by angiotensin-converting enzyme (ACE), leading to sodium retention, vasoconstriction, and cardiovascular damage; and the alternative pathway, where ACE-2 converts Ang-2 to Ang 1-7, interacting with Mas receptors to provide vasodilatory and anti-inflammatory effects (23–25).

As formerly described, RAS is crucial for BP homeostasis, especially in the kidney, where it regulates perfusion during volume depletion and inhibits fluid overload states (22). However, dysregulation of this system contributes to SAH through mechanisms such as increased sodium retention, altered natriuresis, endothelial dysfunction, vascular injury, and enhanced salt sensitivity.

Moreover, the SNS is crucial in developing SAH as it interacts with baroreceptors, immune responses, and renal function to regulate BP (22). Baroreceptors in the carotid sinus detect BP changes and communicate with the brain to modulate SNS activity. It has been observed that hypertensive subjects show increased sympathetic outflow and decreased parasympathetic activity. This hyperactivity is more pronounced in individuals with obesity, metabolic syndrome, kidney disease, and a family history of SAH (22).

The SNS activation effects are mediated by adrenergic neurotransmitters named catecholamines (norepinephrine, epinephrine, dopamine), which cause vasoconstriction (26). In SAH, SNS overactivation leads to renal dysfunction through increased sodium retention and renal fibrosis, resulting in salt sensitivity. Catecholamine-induced hypertension causes interstitial injury and impairs sodium excretion, worsening BP elevation with a high-salt diet. Moreover, SNS activation of α_1_-adrenergic receptors induces endothelial dysfunction, vasoconstriction, vascular smooth muscle proliferation, and arterial stiffness, perpetuating high BP (22, 26).

Furthermore, evidence suggests a bidirectional relationship between the SNS and immune system in SAH. Increased sympathetic activity activates T-lymphocytes and causes vascular inflammation, while proinflammatory cytokines like IL-6 stimulate SNS activation, sustaining hypertension (27). Also, chronic SNS overactivity can desensitize β_2_-adrenoceptors on lymphocytes, altering immune function. Although direct human evidence is limited, studies in rheumatoid arthritis patients show heightened sympathetic activity, especially in hypertensive individuals, reinforcing the link between inflammation and noradrenergic activity activation (28).

In the subsequent paragraphs, we will emphasize the role of inflammation in the development of SAH.

### 3.1 Role of inflammation in SAH

Although SAH has historically been attributed to hemodynamic factors, contemporary research has underscored the significant role of immune-driven inflammation in the progression of the disease. Experimental models have indicated the accumulation of immune cells, particularly T cells and monocytes/macrophages, within the vasculature, kidneys, and brain. These immune cells contribute to the increase in vascular permeability and facilitate the release of ROS, nitric oxide (NO), cytokines, and metalloproteinases, which are critical in driving vascular remodeling. Cytokines promote the formation of the neo-intima layer, reducing the lumen diameter of resistance vessels while increasing vascular stiffness and resistance (20, 22). Additionally, they affect renal tubular function by enhancing the local secretion of angiotensinogen and Ang-2, leading to sodium retention and fluid overload, ultimately elevating BP (29).

The cytokines that play a crucial role in the development of SAH are mostly pro-inflammatory, such as IL-17, TNF-α, gamma interferon (IFN-γ), and IL-1β (29, 30). Together, these cytokines lead to endothelial dysfunction, immune activation, and renal impairment, highlighting the inflammatory pathogenesis of hypertension. IL-17, mainly released by CD4^+^ and CD8^+^ T cells, triggers the expression of CCL-2 in a nicotinamide adenine dinucleotide phosphate (NADPH) oxidase-dependent manner, which results in chronic oxidative stress and T cell infiltration in vascular smooth muscle (31). IL-6, secreted by monocytes, macrophages, and DC, polarizes CD4^+^ T cells and promotes sodium and water retention, further exacerbating SAH. TNF-α stimulates the Nuclear Factor kappa-light-chain-enhancer of activated B cells (NF-κB) and NADPH oxidase, thereby enhancing the release of chemokines and adhesion molecules in blood vessels, which contributes to microvascular remodeling, sodium retention, and diminished NO production (29, 30). IFN-γ, generated by Th1 cells, is associated with angiotensinogen expression, and its deficiency has been shown to reduce renal fibrosis and increase glomerular filtration rate (32). Lastly, IL-1β, released by monocytes, T cells, and neutrophils, leads to macrophage polarization toward the pro-inflammatory M1 subtype, resulting in a substantial release of IL-6 (29, 30).

It has recently been proposed that SAH constitutes a disorder influenced by neuroinflammation (33–35), partially resulting from chronic SNS activation that stimulates microglia, which release pro-inflammatory cytokines such as IL-1β, TNF-α, IL-6, and IFN-γ, thereby worsening high BP levels due to heightened sympathetic nerve activity. Additionally, a compromised blood-brain barrier (BBB) creates a positive feedback loop, enabling circulating inflammatory molecules, Ang-2, and gut-derived metabolites to infiltrate autonomic brain regions, exacerbating neuroinflammation and affecting autonomic regulation dysfunction (29, 36).

Overall, SAH is not merely a hemodynamic disorder, but a complex condition driven by inflammatory and neuroimmune mechanisms. Inflammation is essential in sustaining elevated BP and contributing to target organ damage. Moreover, it represents a common pathological link between MASLD and SAH, suggesting that it may be a crucial connection between these two conditions. Therefore, in the following sections, we aim to present evidence that explains how inflammation bridges MASLD and SAH, highlighting its role in their pathophysiology and potential therapeutic implications.

## 4 Inflammatory crosstalk between MASLD and SAH

As mentioned in the former sections, inflammation is a widely recognized mechanism linking MASLD and SAH. In MASLD, damage to hepatocytes and lipotoxicity provoke an immune reaction that releases pro-inflammatory cytokines such as CXCL9, CXCL10, VCAM-1, IL-6, TNF-α, IL-1β, and IL-17A (37, 38). These cytokines might spread beyond the liver into the bloodstream, potentially leading to a pro-inflammatory systemic state that impacts distant organs, including those that regulate blood pressure. This inflammatory microenvironment is believed to affect the activity of the RAS and SNS—two essential regulators of vascular tone and BP (37, 39, 40).

Obesity is associated with a state of low-grade chronic inflammation (LGCI), characterized by ongoing immune activation and elevated levels of inflammatory mediators in the circulation (41). In adipose tissue, enlarged adipocytes release chemokines and adipokines that attract and activate immune cells, creating a pro-inflammatory environment that influences not only metabolism but also other physiological processes. This inflammatory pathway contributes to all features of MASLD. Additionally, obesity-related metaflammation disrupts endothelial balance by decreasing nitric oxide (NO) availability and increasing levels of asymmetric dimethylarginine (ADMA) and endothelin-1 (ET-1), which promote vasoconstriction, vascular remodeling, and hypertension (42). Therefore, obesity plays a key role in linking MASLD with systemic arterial hypertension (SAH) by heightening inflammation across organ systems and supporting the development of immunometabolic and vascular dysfunction. Its high prevalence among MASLD patients highlights its central role in the pathophysiological interplay between hepatic and vascular diseases (41–43). This underscores the notion that targeting obesity-associated inflammation could be critical not only for managing MASLD but also for preventing or mitigating its vascular complications.

Concurrently, diminished levels of anti-inflammatory cytokines, specifically IL-10, are observed mainly in patients with both MASLD and SAH, thereby further skewing the balance towards a pro-inflammatory state that may amplify the sensitivity of these regulatory systems (44). A positive correlation has been identified between ACE levels and Th2 cytokines IL-4 and IL-13, indicating that these signals may also regulate RAS activity (8). Furthermore, hepatic expression of IL-13 was notably elevated in individuals diagnosed with morbid obesity, MASLD, and SAH when compared to those without SAH. Although the potential role of Th2 cytokines in regulating hepatic ACE remains poorly defined, emerging evidence indicates that IL-4 and IL-13 may modulate its expression via indirect transcriptional mechanisms. In murine and pulmonary models, IL-13 has been shown to increase the transcription factor EGR1 (45), which mediates processes such as inflammation, fibrosis, and apoptosis by regulating genes including TGF-β1, caspases, and various chemokines. ChIP-seq data also reveal *IL4R* as a downstream target of EGR1 (46), and EGR1 itself is capable of binding to and activating the *IL4* promoter in T cells, thereby reinforcing type 2 immune responses.

Additionally, IL-4 and IL-13 activate signaling pathways involving STAT6 and STAT3 (47). Although these regulatory cascades have not been directly validated in hepatic models, they imply a plausible immunometabolic connection between Th2 cytokines and the transcriptional regulation of RAS components. Although further mechanistic research is necessary, this link suggests that chronic type 2 inflammation may promote local RAS activation in the liver, potentially exacerbating fibrosis and systemic vascular issues. In this scenario, IL-4 and IL-13 could act as a mechanistic link between MASLD and systemic arterial hypertension by connecting immune dysregulation to changes in RAS-related gene expression.

If liver-produced cytokines are released into the portal circulation, they may reach the systemic periphery and affect vascular tone (48), thereby representing a novel immunohepatic mechanism in the pathogenesis of hypertension in MASLD patients.

Evidence suggests that hepatic inflammation in MASLD could evolve into a systemic issue (39). This progression is influenced by traditional inflammatory mediators and targeted immunological signals that engage with vascular regulatory systems, which may contribute to the development of SAH. However, it is equally possible that the inflammatory signals are initiated reversely. Thus, further investigations should examine these hypotheses.

In the following paragraphs, we will analyze the role of cytokines in greater detail, which are considered critical in the inflammatory bridge between MASLD and SAH.

### 4.1 CXCL9

**Figure 1:**
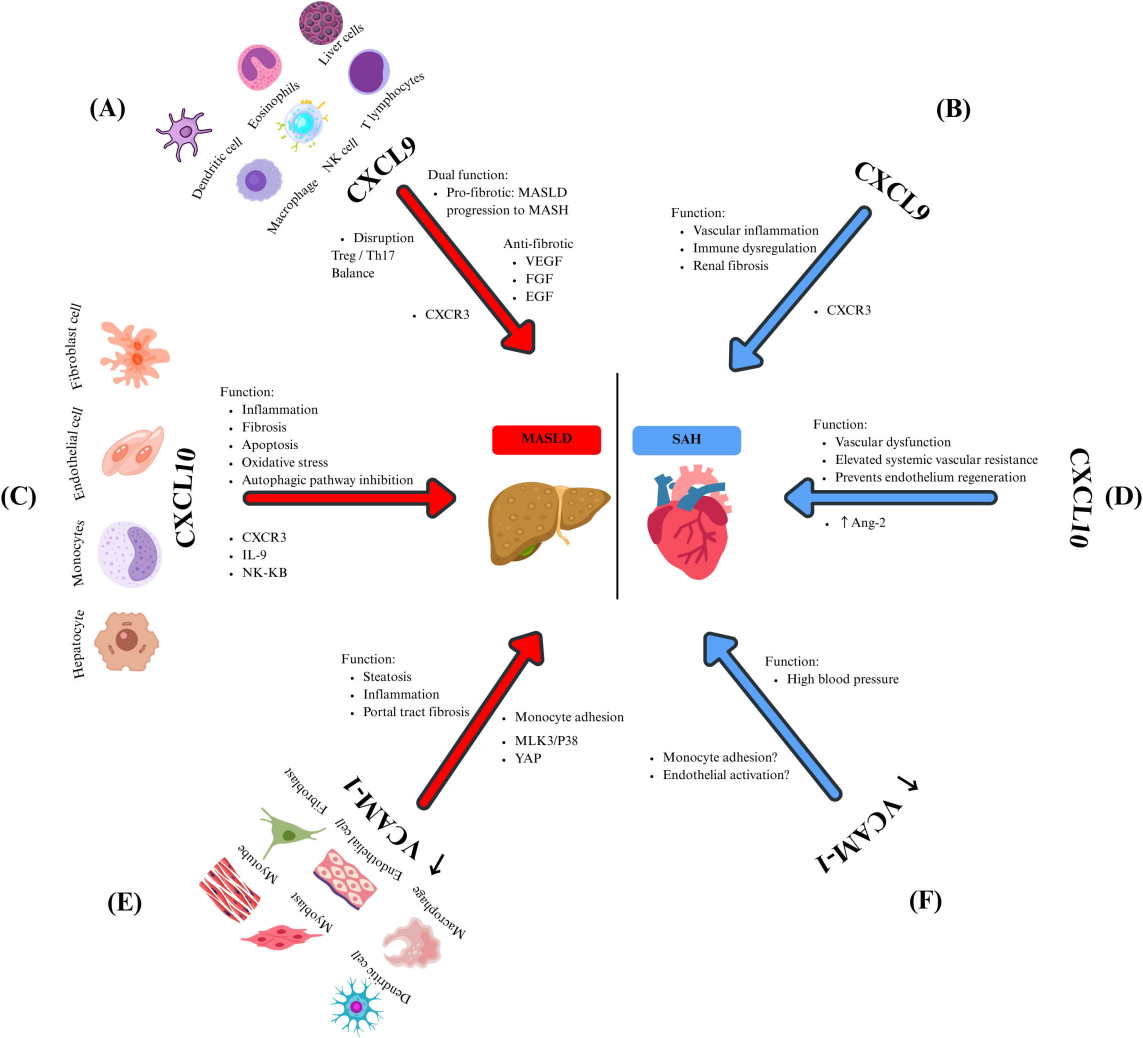
The effects of chemokines and their mediators in MASLD and SAH. CXCL9 is expressed by several immune cells, and it is related to **(A)** MASLD through pro-fibrotic (disrupting the Treg/Th17 balance) and anti-fibrotic (through the inhibition of heparan sulfate proteoglycan-mediated signaling of multiple endothelial growth factors) functions. Meanwhile, in SAH **(B)**, the CXCL9-CXCR3 axis promotes vascular inflammation, immune dysregulation, and renal fibrosis. Hepatocytes, monocytes, endothelial cells, and fibroblasts secrete CXCL10. Its overexpression in MASLD **(C)** is related to inflammation, fibrosis, oxidative stress, and inhibition of the autophagic pathway by promoting signaling pathways such as IL-9 and NF-kB. On the other hand, in SAH **(D)**, CXCL10 promotes vascular dysfunction, elevated systemic vascular resistance, and prevents the regeneration of the endothelium by Ang-2 regulation of its expression. VCAM-1 is produced by dendritic cells, myoblasts, myotubes, macrophages, endothelial cells, and fibroblasts. In the context of MASLD **(E)**, it causes steatosis, inflammation, and portal tract fibrosis primarily through the regulation of monocyte adhesion. It is significantly overexpressed in lipotoxic situations, which are controlled by the proteins MLK3/p38 and YAP. In patients with SAH **(F)**, elevated VCAM-1 levels were observed, and these levels correlate with increased BP. However, the underlying mechanism remains unclear. It is still hypothesized that it may involve monocyte adhesion and endothelial activation.

CXCL9 is a low-molecular-weight chemokine that belongs to the CXC chemokine family, playing a role in immunoregulatory and inflammatory processes. Its primary function is to recruit leukocytes to sites of inflammation, and it is secreted by various cell types, including T lymphocytes, NK cells, dendritic cells, macrophages, eosinophils, and non‐immune cells, such as hepatic cells (49). Additionally, it helps modulate the activation and proliferation of hepatocytes, stellate cells, and endothelial cells in the liver (Sahin, Trautwein, and Wasmuth, 2010). Semba et al. found high local expression of CXCL9 in the liver of a fatty liver mouse model with non-alcoholic steatohepatitis (50), suggesting a near correlation between steatohepatitis development and elevated local CXCL9 levels. Antibody-mediated neutralization of CXCL9 during established obesity conferred protection from hepatocellular damage (51), suggesting that activating the CXCL9 axis may represent an essential hallmark of MASLD progression. CXCL9 plays a key role in inflammation, collagen deposition, and tissue remodeling and is involved in the pathogenesis and complications of MASLD (**Figure 1A**) (52). This is demonstrated by Wang and collaborators, who found that CXCL9 was highly expressed in patients with MASH, correlating with liver injury and complications of liver disease in humans (53).

Furthermore, CXCL9 has been identified as a prognostic marker in patients with liver cirrhosis. Elevated levels of this chemokine have been reported to be associated with shorter survival in cirrhotic patients (54). Additionally, CXCL9 exacerbates the progression of MASLD by disrupting the balance between Treg/Th17 cells (55). Therefore, antagonizing this chemokine could represent a therapeutic approach to ameliorate acute and chronic fibrotic liver damage.

Contrary to expectations, some authors have reported some antifibrotic properties of CXCL9. Wasmuth and colleagues found that this chemokine exerted antifibrotic effects *in vitro* by suppressing collagen in mice and human liver cells (56). Similarly, Sahin and colleagues identified direct angiostatic and antifibrotic effects of the CXCR3 ligand CXCL9 in a model of experimental liver fibrosis (57). However, the observed antifibrotic and antiangiogenic effects of CXCL9 may occur through the inhibition of signaling mediated by heparan sulfate proteoglycans involves various endothelial growth factors, including vascular endothelial growth factor (VEGF), fibroblast growth factor (FGF), and endothelial growth factor (EGF) (**Figure 1A**) (58).

A likely explanation for the reported elevation of CXCL9 in patients with MASLD might be positive feedback and upregulation of this protein to counteract the fibrotic milieu within damaged liver cells. However, the elevation of CXCL9 in patients with MASLD might also point to this chemokine as the initial activator of many pathways that ultimately lead to fibrosis and culminate in the development and progression of MASLD.

The function of CXCL9 in regulating BP remains not fully understood. Nevertheless, evidence indicates that the CXCL9/CXCR3 axis plays a multifaceted role in maintaining renal and vascular homeostasis (59). Research has shown that Ang 2-stimulated CXCR3-deficient mice experience elevated blood pressure due to increased AT1R levels, suggesting that the absence of CXCR3 may render them more susceptible to salt-sensitive hypertension (60). These mice display higher mortality rates and worsening interstitial fibrosis despite less pronounced inflammatory cell infiltration (61). The role of CXCR3 in fibrosis and organ damage seems paradoxical, as CXCR3 is upregulated in cases of progressive renal fibrosis (**Figure 1B**) (59). Conversely, disrupting the CXCL-9/CXCR3 pathway has been shown to improve kidney function and reduce the presence of activated macrophages and T cells in murine models, indicating a complex and context-specific function of this pathway in renal inflammation and fibrosis. Additionally, patients with SAH show elevated serum levels of CXCL9 compared to normotensive controls. Moreover, these patients presented increased counts of immunosenescent CD8+ T cells, indicating that CXCL9 could play a role in immune dysregulation and vascular inflammation in hypertension (62).

CXCL9 is a multifunctional chemokine with a central role in immune regulation, inflammation, and tissue remodeling, particularly within the liver. It is related to the pathogenesis and progression of MASLD, as it has been reported that elevated hepatic and serum levels correlate with liver injury, fibrosis, and poor prognosis. However, its role remains complex and context-dependent, as some studies report antifibrotic and angiostatic effects, possibly as a compensatory response to liver damage. Beyond the liver, the CXCL9/CXCR3 axis also appears to be involved in renal and vascular homeostasis, with implications for hypertension and systemic inflammation. While CXCL-9 is a promising biomarker and potential therapeutic target, further research is needed to clarify its dualistic roles and determine how best to modulate its activity in liver and vascular diseases.

### 4.2 CXCL10

CXCL10 is a critical chemokine secreted by many cell types, including monocytes, endothelial cells, and fibroblasts, in response to IFN-γ. The function of CXCL10 has been attributed to pleiotropic effects, including stimulation of monocytes, natural killer cells, and T-cells migration, and modulation of adhesion molecule expression. In the liver, hepatocytes are a significant source of CXCL10, which exerts and perpetuates liver inflammation, playing a crucial role in the pathogenesis of MASLD (63). However, in various types of liver injury, CXCL10 is secreted by hepatic cells in areas of lobular inflammation (64).

By binding to CXCR3, CXCL10 recruits T lymphocytes and macrophages to the liver parenchyma, promoting inflammation, apoptosis, and fibrosis (55) through the direct induction of the proinflammatory cytokine IL-9 in a liver fibrosis model (65). Additionally, a systematic review by Pan and colleagues found that CXCL10 triggers oxidative stress, fibrosis, and inflammation in MASH by activating the NF-κB pathway through activated B-cells (**Figure 1C**). This activation stimulates macrophages, leading to fibrosis and liver injury in MASLD (66).

It has been proposed that high CXCL10 levels may be involved in MASH due to the inhibition of the autophagic pathway (67). In an *in vitro* study, it was demonstrated that high levels of CXCL10 reduced the accumulation of phagocytic proteins and enhanced the degradation of autophagic proteins in liver cells, ultimately contributing to the onset and progression of MASH (67). Additionally, CXCL10 effects are mediated by KC, as demonstrated by the induction of inflammation in the liver and the stimulation of inflammatory monocyte infiltration, which produces CXCL10 and limits liver sinusoidal endothelial cell permeability (**Figure 1C**) (68).

Elevated CXCL10 levels also play a significant role in the pathophysiology of SAH. Antonelli and colleagues observed that patients with untreated essential hypertension showed notably higher CXCL10 serum levels than healthy individuals, suggesting that this chemokine might be involved in the pathophysiology of SAH (69). Additionally, increased Ang-2 levels are a key marker of SAH’s pathophysiology, and a close association between elevated levels of this RAS peptide and high levels of pro-inflammatory chemokines has been reported (70). In this sense, it is known that Ang-2 promotes the expression of chemokines, such as CXCL10, in various cell types, including fibroblasts, endothelial cells, and smooth muscle cells. In response, monocytes, macrophages, and T-cells migrate into the adventitia and periadventitial fat. This phenomenon is suggested to maintain vascular dysfunction, elevate oxidative stress, and lead to fibrosis, all contributing to increased BP (**Figure 1D**) (71). Campanella and collaborators found that CXCL10 can inhibit endothelial cell proliferation and, thus, prevent the endothelium regeneration needed to remove chronic alterations in the structure of blood vessels, contributing, in this manner, to perpetuate elevated systemic vascular resistance in hypertensive subjects (72). These observations support the notion that CXCL10 may function as a common mediator of immune activation and tissue injury in both hepatic and systemic settings, potentially in the inflammatory and vascular interactions that connect MASLD and SAH.

### 4.3 VCAM-1

VCAM-1 is a cell surface sialoglycoprotein expressed by activated endothelial cells, tissue macrophages, DC, bone marrow fibroblasts, myoblasts, and myotubes. Its primary function consists of mediating adhesion to vascular endothelium cells and transmigration of lymphocytes, monocytes, eosinophils, and basophils into the subendothelial space during inflammation (73).

Recently, it has been associated with the onset of chronic liver diseases and fibrosis, which is upregulated and promotes MASH by mediating monocyte adhesion to the liver sinusoidal endothelial cells. In this context, VCAM-1-neutralizing monoclonal antibodies or pharmacological inhibition have provided a successful attenuation of diet-induced steatohepatitis in mouse models via reducing the proinflammatory monocyte hepatic population, which highlights the key role of VCAM-1 in the pathogenesis of chronic liver diseases (**Figure 1E**) (74). Lefere and colleagues studied endothelial dysfunction in MASLD patients, identifying VCAM-1 as an independent predictor of significant fibrosis in this population (75). Furthermore, Halima et al. investigated the interaction between VCAM-1 and liver fibrosis within the serum of patients diagnosed with chronic liver diseases. Their findings indicate that elevated levels of this molecule significantly predict unfavourable outcomes in individuals suffering from MASLD (76).

The previous results that highlight the link between VCAM-1 and MASLD can be clarified by understanding that VCAM- 1 prompts monocytes to migrate to the liver through fenestrae, possibly triggering a pro-inflammatory response, as noted by Furuta and colleagues. Their study demonstrated that lipotoxic stress in MASLD elevates VCAM-1 levels in liver sinusoidal endothelial cells due to the activation of the Mixed-Lineage Kinase 3 (MLK3) and the p38 MAPK pathway (MLK3/P38). This activation subsequently enhances the transmigration of pro-inflammatory cells, worsening the hepatic condition (**Figure 1E**). This finding is bolstered by evidence indicating that blocking VCAM-1 prevents the adhesion and migration of monocytes across the endothelium, thus hindering the progression of MASLD (74).

Toxic lipid levels caused high expression of VCAM-1, which activated HSC *via* the yes-associated protein-1 (YAP-1) signaling pathway. Further experiments confirmed this data, showing that the specific deletion of *VCAM1* in liver endothelial cells resulted in a better liver fibrosis profile in *in vitro* models (77). Recently, it has been noted in a rat model that increased VCAM-1 levels in hepatic endothelium facilitate the recruitment of inflammatory macrophages, driving vascular remodeling and leading to portal tract fibrosis (**Figure 1E**) (78).

The role of VCAM-1 in developing SAH has also been studied (79, 80). Significantly higher plasma levels of VCAM-1, as part of the hypertension pathophysiology, have been previously observed in many research studies, suggesting a close interconnection (81, 82). In a rat model, Ng and his team identified a positive correlation between increased VCAM-1 levels and elevated blood pressure. They examined the expression of this chemokine in the aortic tissue of rats, discovering that those with higher blood pressure exhibited more significant levels of VCAM-1 expression (83). Evidence suggests a connection between SAH development and elevated VCAM-1 levels; however, the underlying immunopathological mechanisms remain under investigation. A proposed mechanism focuses on the chemotactic properties of VCAM-1 in the endothelium. VCAM-1 is crucial for monocyte adhesion and migration to vascular endothelium, which, in chronic inflammation as seen in SAH patients, is linked to perpetuating hypertension (79). Yin and colleagues demonstrated that inhibiting VCAM-1 with a monoclonal antibody reduces monocyte adhesion and infiltration into the endothelium, thereby protecting against arterial hypertension and dysfunction caused by Ang-2 in a mouse model (79) (**Figure 1F**). According to Palomo and colleagues, it has been suggested that chronic endothelial activation during SAH leads to a direct overexpression of VCAM-1. They initially established that sVCAM‐1 levels are elevated in hypertensive patients compared to those with normal blood pressure. They then suggested that SAH might boost the production of cell adhesion molecules, likely due to endothelial activation (84).

The data indicate that VCAM-1 is critical in MASLD and SAH due to its repetitive links to hepatic inflammation, fibrosis, vascular dysfunction, and increased BP. However, the exact role of VCAM-1 in connecting these two disorders is still uncertain. It remains unclear whether VCAM-1 expression begins in the liver or the vascular endothelium, and whether its systemic increase in one area affects the onset or progression of the other. More research is necessary to determine whether VCAM-1 is merely a coincidental indicator of chronic inflammation in MASLD and SAH, or if it is a key mechanism driving the immunometabolic relationship between these two conditions.

### 4.4 TNF-α

**Figure 2:**
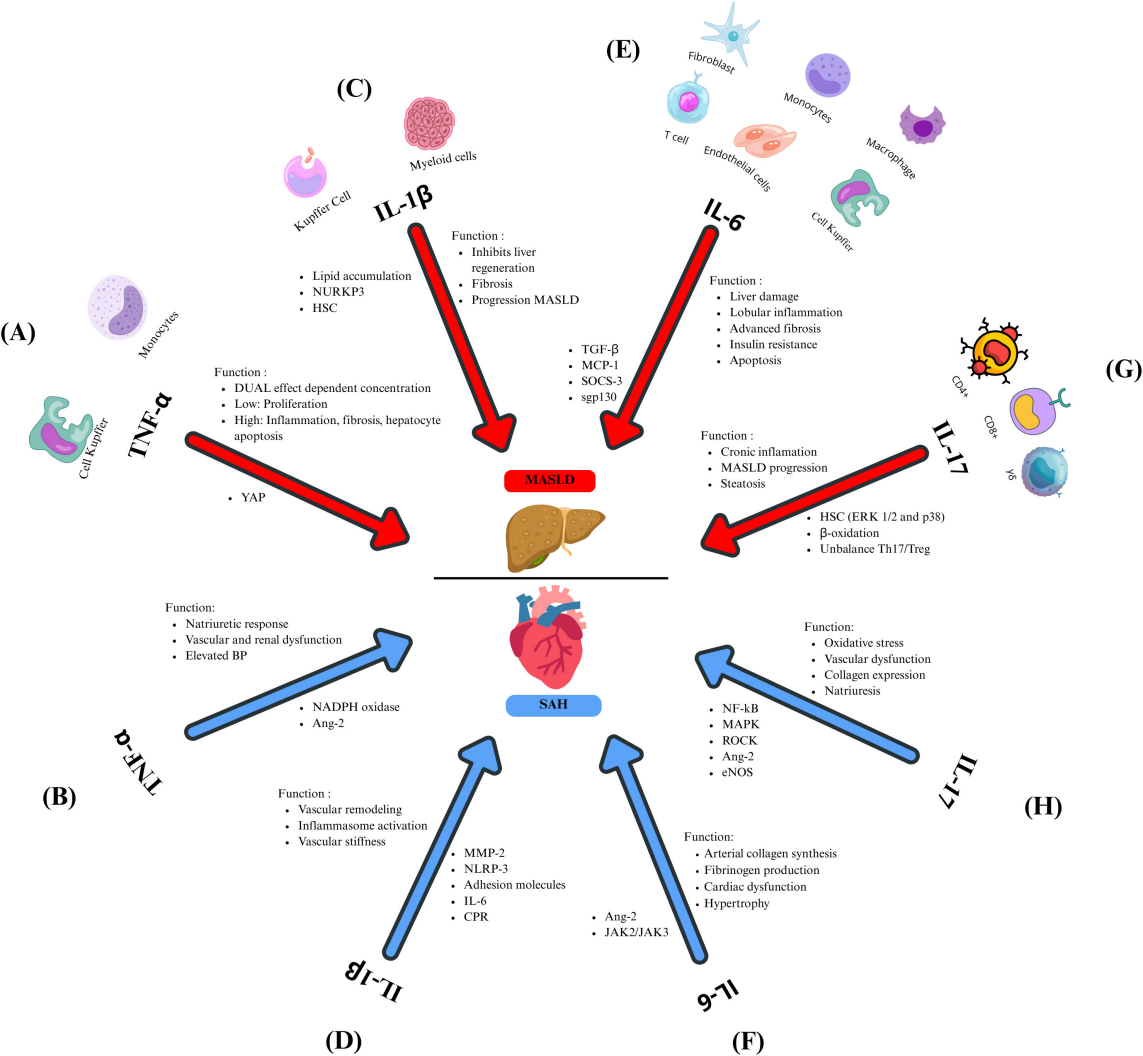
Inflammatory cytokines related to MASLD and SAH. TNF-α plays a dual role in MASLD **(A)**, influenced by its concentration. At low levels, this cytokine promotes the proliferation of hepatocytes, whereas elevated concentrations result in inflammation, fibrosis, and apoptosis of hepatocytes. Both mechanisms are mediated through the modulation of YAP. In SAH **(B)**, TNF-α modulates the natriuretic response and contributes to vascular and renal dysfunction and elevated BP, contributing to SAH by regulating NADPH oxidase and Ang-2. **(C)** IL-1β inhibits liver regeneration, promotes fibrosis, and favors the progression from MASLD to MASH, allowing lipid accumulation, neutrophil recruitment, and the activation of HSC. In MASLD, this cytokine is regulated by NURKP3. **(D)** Through MMP-2, IL-1β promotes vascular remodeling leading to vascular stiffness, atherosclerosis contributes to the increment of NLRP3, which leads to IL-1β overexpression, and this cytokine induces the expression of adhesion molecules, IL-6, and CPR. **(E)** IL-6 is involved in liver damage, lobular inflammation, advanced fibrosis, insulin resistance, and apoptosis by regulating inflammatory markers such as TGF-β, MCP-1, and SOCS-3. The IL-6/gp130 axis promotes the progression to MASH. **(F)** IL-6 favors arterial collagen synthesis, fibrinogen impairment, cardiac dysfunction, and hypertrophy by the Ang-2 and JAK2/JAK3 regulation during SAH. **(G)** In MASLD, IL-17 contributes to chronic inflammation, steatosis, and progression from MASLD to MASH by activating HSC by Erk1/2 and p38, inhibiting the β-oxidation. It favors the imbalance between Th17 and Treg. In SAH **(H)**, IL-17 promotes oxidative stress, vascular dysfunction, collagen expression, and regulates natriuresis by regulating inflammatory signaling pathways such as NF-kB, MAPK, ROCK, Ang-2, and eNOS.

TNF-α is a proinflammatory cytokine produced by monocytes and macrophages, with roles in immune response, apoptosis, and inflammation. It acts through two receptors, TNFR-1 and TNFR-2, found in nearly all cell types, including liver cells (85). In MASLD, KCs respond to hepatic injury, releasing TNF-α and attracting inflammatory monocytes. For decades, it has been established that TNF-α contributes to liver inflammation, fibrosis, hepatocyte apoptosis, and disease progression. However, it was recently proposed that the function of TNF-α in the liver may depend on its concentration. Zhao and colleagues demonstrated in a *TNF* ^−/−^ murine model treated with different concentrations of this cytokine that low levels of TNF-α reduce plasma indicators of liver injury, including alanine aminotransferase (ALT) and aspartate aminotransferase (AST), promote hepatocyte growth by modulating Yap activity protein, which is responsible for balancing cell proliferation and death in organs. However, these damage markers increased in a dose-dependent manner with TNF-α concentration, along with the phosphorylation and inactivation of Yap (**Figure 2A**) (86). Inhibition or deletion of TNF-α and/or its receptors in animal models enhances liver health by reducing fat accumulation and lowering ALT levels, decreases inflammation through reduced levels of IL-1β and IL-6, and improves insulin sensitivity by decreasing the activity of factors like JNK and NF-kB while exhibiting higher levels of adiponectin (87, 88). Clinical studies confirm elevated levels of TNF-α and its receptors in the liver and serum of patients with MASLD, which are linked to the severity of the disease (89).

Due to its systemic effects, dysregulation of TNF-α is associated with hepatic inflammation and other conditions characterized by chronic inflammation. One such condition is SAH, where TNF-α influences renal function by regulating hemodynamic and excretory processes, thus playing a role in the natriuretic response. This cytokine is implicated in vascular dysfunction and elevated BP. TNF-α contributes to the development of SAH by activating NADPH oxidase in polymorphonuclear leukocytes (PMN), producing oxidative stress. Moreover, TNF-α enhances Ang-2 signaling, driving renal and vascular dysfunction (**Figure 2B**) (90, 91). TNF-α inhibition with etanercept in salt-sensitive rat models diminishes the development of hypertension (92). Similarly, in patients with resistant hypertension, blocking TNF-α with infliximab reduces blood pressure (93), underscoring the pivotal role of this cytokine in the SAH pathophysiology. TNF-α is critical in MASLD and SAH, regulating inflammation and metabolic mechanisms. Therefore, targeting TNF-α could be a promising strategy for treating these chronic conditions.

### 4.5 IL-1β

IL-β is a pro-inflammatory cytokine with pleiotropic functions involved in inflammation and acquired immunity. It is produced as a precursor named proIL-1β, which requires enzymatic processing for activation, mediated by caspase-1 and the inflammasome. IL-1β is constitutively present in cells; however, its expression is induced by inflammatory stimuli such as Toll-like receptors (TLR) ligands or the release of alarmins. Following inflammasome activation, this cytokine is produced by resident macrophages and other myeloid cells (94). In the liver, KCs are responsible for producing IL-1β and expressing its receptor IL-1Ra. In liver failure and regeneration models, it was found that IL-1β inhibits liver regeneration while IL-1Ra promotes it (95). In animal models, the IL-1Ra deletion drives severe steatosis and fibrosis, highlighting the need for a balance between these molecules (96). IL-1β favors the progression of MASLD, allowing lipid accumulation in hepatocytes and increasing inflammation by the rise of neutrophil recruitment through the expression of intracellular adhesion molecule (ICAM-1) (97). Reducing IL-1β levels diminishes lipid accumulation in the liver (98). The NOD-, LRR-, and pyrin domain-containing protein 3 (NLRP3) inflammasome, a crucial component in liver inflammation and fibrosis, triggers the activation of IL-1β and IL-18. This activation is associated with disease progression in both animal studies and human cases of MASH (99, 100). IL-1β also contributes to advanced steps in the disease, specifically in fibrosis, by stimulating HSC, increasing tissue inhibitor of metalloproteinases 1 (TIMP-1) production, and preventing extracellular matrix degradation (**Figure 2C**).

Beyond liver disease, IL-1β-driven inflammation increases CVD risk. While IL-1β inhibition via canakinumab did not lower blood pressure in the CANTOS trial, it reduced cardiovascular events (101). Mechanisms by which IL-1β may drive hypertension include metalloproteinase 2 (MMP-2) activation, promoting vascular remodeling, sustained inflammasome activation, and extracellular matrix alterations that increase vascular stiffness (102). Atherosclerosis activates NLRP3 via cholesterol crystals and oxidized low-density lipoproteins (LDL) (**Figure 2D**), amplifying IL-1β release and cardiovascular inflammation (103). IL-1β also upregulates adhesion molecules and IL-6, contributing to systemic inflammation and elevated C-reactive protein levels (104).

IL-1β is a central mediator in the pathogenesis of MASLD and SAH. It promotes liver steatosis, fibrosis, and insulin resistance while contributing to systemic and vascular inflammation that underlies various forms of hypertension. Targeting IL-1β or its receptor presents a potential therapeutic strategy for reducing inflammation and preventing disease progression in hepatic and cardiovascular contexts.

### 4.6 IL-6

IL-6 is a key pro-inflammatory cytokine produced by various cell types, including fibroblasts, monocytes, macrophages, T cells, endothelial cells, and KC, which contributes to the acute-phase response, host defense, and liver regeneration (105). IL-6 significantly contributes to the pathogenesis of MASLD. Both wild-type and *IL6*-deficient mice (*IL6* ^-/-^), when fed with a methionine and choline-deficient diet (MCD), developed steatohepatitis and experienced weight loss. Nevertheless, the *IL6* ^-/-^ mice showed lower serum concentrations of liver damage markers, including ALT, and decreased blood glucose, cholesterol, and triglyceride levels. Despite developing similar levels of steatohepatitis as wild-type mice, the *IL6* ^-/-^ mice exhibited less lobular inflammation. Furthermore, they showed lower levels of inflammatory markers, such as TGF-β and monocyte chemoattractant protein-1 (MCP-1). These findings suggest that IL-6 plays a crucial role in hepatic inflammation (106) IL-6 also promotes systemic IR through the upregulation of the suppressor of cytokine signaling 3 (SOCS-3). Elevated hepatic and serum IL-6 levels correlate with more significant inflammation, fibrosis, and insulin resistance in patients with MASH (**Figure 2E**) (107, 108).

It has also been reported that IL-6 receptor (IL-6R) concentrations decline in advanced stages of MASLD. They are associated with increased apoptosis in hepatocytes and leukocytes, suggesting IL-6R as a potential biomarker of MASLD progression (109) and in patients with obesity and MASH, increased soluble glycoprotein 130 (sgp130), which belongs to the IL-6 family, correlate with glycated hemoglobin (HbA1c), liver stiffness, and advanced fibrosis, indicating that glucose metabolic dysregulation may intensify IL-6 activity and worsen MASLD (110). In animal models, blocking IL-6/gp130 signaling prevents the progression to MASH. It reduces hepatic injury markers such as ALT and apoptosis despite increased lipid accumulation in the liver (**Figure 2E**), implying a dual role in protecting against MASH but enhancing inflammation (111).

Beyond liver disease, IL-6 is an established cardiovascular risk marker. It promotes arterial collagen synthesis and fibrinogen production and has been associated with coronary endothelial dysfunction (112) and atherosclerosis (113). In animal models, IL-6 administration led to cardiac dysfunction, hypertrophy, and fibrosis, effects observed independently of BP (114, 115). IL-6 inhibition using anti-interleukin-6 receptor antibody (MR16-1) improved left ventricular remodeling (116). Chronic infusion of growing doses of Ang-2 potentially stimulates the release of IL-6 through the renal Janus Kinases (JAK2/JAK3) signaling pathway, increasing the mean BP (117) (**Figure 2F**).

The relationship between IL-6, MASLD, and SAH has been little explored, but experimental evidence indicates a probable connection. The inflammatory properties of IL-6 could be key in the transition from MASLD to SAH, and additional research is required to elucidate this interconnection.

### 4.7 IL-17

IL-17 is a proinflammatory cytokine produced by various immune cells in response to IL-1β and IL-23. The inflammatory effects triggered by IL-17 are typically controlled by regulatory T cells (Treg) and anti-inflammatory cytokines such as IL-10 and TGF-β (118). IL-17A plays a role in chronic low-grade inflammation, facilitating the progression from hepatic steatosis to MASH and liver fibrosis (119). In an HFD animal model, the inhibition of IL-17A reduces liver damage and inflammatory infiltration. Moreover, in hepatocytes in cultures exposed to FFA and IL-17A, this cytokine promoted steatosis by disrupting insulin signaling. Additionally, IL-17A activates HSC via extracellular signal-regulated kinases (ERK1/2) and p38 pathways, contributing to fibrosis (120). The exacerbated hepatic steatosis produced by IL-17A is caused by inhibiting fatty acid β-oxidation and promoting TG accumulation (121). Using therapeutic strategies targeting the IL-17A pathway and drugs that prevent the progression from MASLD to MASH, such as polyunsaturated phosphatidylcholine (PPC) and 3’,3’-diidolylmethane (DIM), restored the immune balance Th17/Treg reducing liver inflammation and the hepatic steatosis (**Figure 2G**) (122, 123). Blocking IL-17A and inhibiting Th17 cell differentiation in mice has demonstrated prevention of MASH and hepatocellular carcinoma (HCC), showcasing the therapeutic promise of IL-17A blockers (124).

Systemically, IL-17A contributes to vascular dysfunction and SAH by promoting oxidative stress, endothelial dysfunction, and vascular stiffness. It triggers inflammatory signaling pathways like NF-κB, p38 mitogen-activated protein kinases (MAPK), and RhoA/Rho-kinase (ROCK) (125), leading to higher collagen expression in vascular smooth muscle cells. Serum levels are increased in patients with SAH, especially in those with diabetes, HIV, or undergoing hemodialysis (126).

In humanized murine models, where the murine immune system was replaced with human hematopoietic stem cells (from bone marrow and liver) and human thymic cells, Ang-2 raises IL-17A levels, leading to vascular and renal inflammation (127). Blocking IL-17A reduces blood pressure, leukocyte infiltration, arterial remodeling, and renal dysfunction (128). IL-17A also regulates sodium transport in the kidneys, influencing natriuresis and blood pressure (129), contributing to endothelial dysfunction via the phosphorylation of the endothelial nitric oxide synthase (eNOS) (130), supporting the hypothesis that IL-17A plays a pathogenic role in the inflammation related to renal hypertension (**Figure 2H**).

IL-17A is an essential inflammatory mediator linking MASLD progression and SAH. It promotes steatosis, fibrosis, and IR in the liver while contributing to vascular inflammation and elevated blood pressure, making it a potential therapeutic target in both hepatic and cardiovascular diseases.

## 5 Discussion

This review describes the most significant features of the intricate and bidirectional interplay between MASLD and SAH, with a focus on inflammation as the central shared pathogenic mechanism. The global burden of MASLD is related to lifestyle factors such as poor diet, physical inactivity, and the obesity pandemic, highlighting its clinical relevance, particularly given its frequent coexistence with cardiovascular and metabolic diseases. Various studies indicate that MASLD and SAH frequently co-occur and exacerbate each other’s development and progression. Clinical data reveal that patients with MASLD have a significantly higher risk of developing SAH, and the severity of liver disease correlates with the degree of BP elevation. On the other hand, individuals with SAH exhibit an elevated risk of progressing to hepatic steatosis and MASH. For this reason, we propose a clinical pathogenic loop mediated by chronic, low-grade inflammation, which could be a mutual driver between these entities.

During MASLD, hepatocyte injury activates a cascade of immune responses that lead to the release of pro-inflammatory mediators. These mediators propagate local hepatic inflammation and enter systemic circulation, where they can modulate vascular function and contribute to the activation of RAS and the SNS, which are key regulators in the pathophysiology of SAH. In contrast, persistent SNS activation in SAH leads to hepatic sympathetic tone and can drive macrophage activation, cytokine release, and progression to liver fibrosis. Ang-2 is another critical effector in the classical RAS, and it is implicated in IR and liver fibrosis.

The cytokine and chemokine milieu in MASLD patients significantly overlaps with what is observed in SAH. In general, TNF-α, IL-1β, IL-6, IL-17A, CXCL9, CXCL10, and VCAM-1 contribute to vascular inflammation and hepatic damage, reinforcing the hypothesis of a unified inflammatory axis. The proinflammatory cytokines TNF-α, IL-1β, IL-6, and IL-17A, along with the chemokines CXCL9 and CXCL10, and the adhesion molecule VCAM-1, play critical roles in the pathogenesis of both MASLD and SAH. Each of these molecules contributes to the inflammatory process in both diseases through various mechanisms and at distinct stages of disease progression. TNF-α, IL-1β, IL-6, and IL-17A regulate inflammation, fibrosis, and metabolic dysfunction. Meanwhile, cytokines such as TNF-α and IL-1β contribute to liver damage in MASLD, while IL-6 and IL-17A influence hepatic and vascular inflammation. Similarly, CXCL9 and CXCL10 promote the recruitment of immune cells, and VCAM-1 facilitates the adhesion of immune cells to endothelial cells in both liver and vascular tissues.

These insights offer a framework for understanding the relationship between MASLD and SAH, although the exact causal pathways remain unclear. It is debated whether systemic hepatic inflammation causes SAH or vice versa (45–47, 131–133). Identifying common inflammatory mediators offers a potential therapeutic opportunity; targeting immune pathways might enhance metabolic, hepatic, and vascular health. Further longitudinal and interventional studies are necessary to determine the sequence and interrupt inflammatory interactions. Approaches such as precision medicine, immune profiling, and metabolic phenotyping could enable more targeted prevention strategies.

Although MASLD and SAH share some inflammatory features, the specific mechanistic pathways connecting these two conditions are not yet clearly understood. Chronic liver inflammation in MASLD may enter the bloodstream, either through the portal-hepatic pathway (48, 134) or as part of obesity-related LGCI (41), triggering immune responses that affect vascular tone and contribute to hypertension. On the other hand, ongoing vascular inflammation in SAH can cause hepatic stress, leading to steatosis and fibrosis. Although these concepts are biologically plausible, they remain theoretical due to the absence of comprehensive experimental evidence supporting them at present.

Cytokines such as TNF-α, IL-6, IL-17A, CXCL9, CXCL10, and VCAM-1 are often elevated in both MASLD and SAH, playing crucial roles in immune cell recruitment, endothelial dysfunction, and fibrogenesis. Significantly, new evidence suggests that Th2 cytokines such as IL-4 and IL-13 can influence parts of the RAS (7), including ACE, through indirect transcriptional pathways (45–47, 132, 133). While this has yet to be fully validated in hepatic models, their potential involvement in immunometabolic crosstalk warrants further investigation. Inflammation seems to be a key common mechanism that can trigger hallmark processes in both diseases, including oxidative stress, IR, endothelial dysfunction, and immune dysregulation. Therefore, a goal for future research is to determine whether these responses originate from shared or interconnected pathways, thereby enhancing our understanding of disease progression and aiding in the identification of common molecular targets for diagnosing, predicting, and treating MASLD and SAH.

## 6 Conclusion

The complex interplay between MASLD and SAH is increasingly understood to be rooted in shared inflammatory mechanisms. Hepatic inflammation in MASLD triggers the release of pro-inflammatory cytokines and chemokines that extend beyond the liver, potentially disrupting vascular homeostasis and promoting hypertension. Conversely, SAH contributes to hepatic dysfunction through hemodynamic stress. This systemic inflammatory environment influences central pathways such as the RAS, creating a vicious cycle that perpetuates both conditions. This review highlights the pivotal role of inflammation-related molecules, including CXCL9, CXCL10, VCAM-1, TNF-α, IL-1β, IL-6, and IL-17A, in the pathogenesis and progression of MASLD and SAH. These molecules contribute to local hepatic inflammation, fibrosis, and metabolic dysregulation and exert systemic effects that promote vascular dysfunction, immune activation, and elevated blood pressure. This bidirectional relationship highlights the importance of investigating anti-inflammatory and immunomodulatory strategies as potential therapeutic approaches to slow the progression of both MASLD and SAH.
